# Human FMO2-based microbial whole-cell catalysts for drug metabolite synthesis

**DOI:** 10.1186/s12934-015-0262-0

**Published:** 2015-06-12

**Authors:** Martina Geier, Thorsten Bachler, Steven P Hanlon, Fabian K Eggimann, Matthias Kittelmann, Hansjörg Weber, Stephan Lütz, Beat Wirz, Margit Winkler

**Affiliations:** acib GmbH c/o Institute of Molecular Biotechnology, Graz University of Technology, NAWI Graz, Petersgasse 14, 8010 Graz, Austria; F. Hoffmann-La Roche Ltd., 4070 Basel, Switzerland; Novartis Pharma AG, 4002 Basel, Switzerland; Institute of Organic Chemistry, Graz University of Technology, NAWI Graz, Stremayrgasse 9, 8010 Graz, Austria

**Keywords:** Flavin monooxygenase isoform 2, *Escherichia coli*, Whole-cell biocatalysis, Drug metabolites, Trifluoperazine, Propranolol, FMO2

## Abstract

**Background:**

Getting access to authentic human drug metabolites is an important issue during the drug discovery and development process. Employing recombinant microorganisms as whole-cell biocatalysts constitutes an elegant alternative to organic synthesis to produce these compounds. The present work aimed for the generation of an efficient whole-cell catalyst based on the flavin monooxygenase isoform 2 (FMO2), which is part of the human phase I metabolism.

**Results:**

We show for the first time the functional expression of human FMO2 in *E. coli*. Truncations of the C-terminal membrane anchor region did not result in soluble FMO2 protein, but had a significant effect on levels of recombinant protein. The FMO2 biocatalysts were employed for substrate screening purposes, revealing trifluoperazine and propranolol as FMO2 substrates. Biomass cultivation on the 100 L scale afforded active catalyst for biotransformations on preparative scale. The whole-cell conversion of trifluoperazine resulted in perfectly selective oxidation to 48 mg (46% yield) of the corresponding *N*^1^-oxide with a purity >98%.

**Conclusions:**

The generated FMO2 whole-cell catalysts are not only useful as screening tool for human metabolites of drug molecules but more importantly also for their chemo- and regioselective preparation on the multi-milligram scale.

**Electronic supplementary material:**

The online version of this article (doi:10.1186/s12934-015-0262-0) contains supplementary material, which is available to authorized users.

## Background

Flavin monooxygenases (FMOs) are flavin containing enzymes that catalyze substrate oxidation at the expense of NADPH and molecular oxygen. FMOs are involved in the phase 1 drug metabolism of compounds containing soft nucleophiles such as sulfur or nitrogen. Among the six known FMO isoforms, the FMO2 isoenzyme—known also as the pulmonary FMO—is expressed in lung tissue [1]. In humans, two alleles have been described: FMO2*1 is the functional full length version that is found in high frequency in Sub-Saharan African populations [2–4] and descendants from these populations in Hispanics [5] whereas the FMO2*2 gene codes for a sequence with a premature stop codon that results in a 64 amino acid C-terminal truncation and yields non-functional protein FMO2X472 [1].

The substrates typically described for human FMO2*1 are sulfur derived compounds such as thioureas [6, 7], thioetherorganophosphates [6], thiazetazone [8] and ethionamide [8, 9]. FMO2 homologues from other mammalian species were shown to catalyze also the *N*-oxidation, e.g. of primary aliphatic alkylamines [10, 11], secondary and tertiary aliphatic alkylamines [10] as well as a few examples of alicyclic amines like nicotine [12] and *N*-deacetyl ketoconazole [13]. Rabbit FMO2 catalyzed the oxidation of prochlorperazine, desmethylperazine and trifluoperazine [10]. These are phenothiazine derived xenobiotics with antipsychotic effects.

In course of the drug discovery and development process, drug metabolites are required for structure elucidation and as analytical references. Furthermore, with the introduction of the metabolites in safety testing (MIST) guidelines by the US Food and Drug Administration in 2008, all metabolites present at >10% of the parent compound in the human metabolism have to be subjected to toxicity studies [14]. In this context two questions arise: Which drug metabolites are formed in the human body and how can these compounds be produced in sufficient amounts? Chemical preparation of authentic drug metabolites often requires multiple steps including several functional group protection and deprotection steps. To circumvent this problem, our aim was the generation of scalable mimicks of single steps of the phase 1 metabolism reactions in vitro. This can be accomplished with heterologously expressed human enzymes in a ready-to-use biocatalyst such as *E. coli*.

The use of whole-cell catalysts is beneficial in many aspects. The enzyme performing the actual biotransformation does not need to be isolated and purified, which saves time and costs. In addition, the half-life of enzyme activity can be prolonged because the cell acts as a protective shield against e.g. shear forces and organic solvents. As many industrially relevant reactions rely on cofactors, whole cells constitute an attractive strategy to regenerate these costly compounds by exploiting the cell metabolism [15].

A prerequisite for productive whole-cell biocatalysts is the efficient expression of the target enzyme. Human flavin monooxygenases (hFMOs) are membrane bound proteins, a class of proteins that are known to be challenging targets for recombinant expression in *E. coli*. Therefore, we investigated—among other critical parameters—the influence of C-terminal truncation of FMO2*1 on the overall biocatalyst activity.

## Methods

### Chemicals and reagents

Ampicillin, benzydamine, ethionamide, (±)-propranolol and trifluoperazine were purchased from Sigma Aldrich (Steinheim, Germany) and acetonitrile (ACN) from J. T. Baker (Deventer, The Netherlands). Isopropyl β-d-1-thiogalactopyranoside (IPTG) was obtained from Biosynth AG (Staad, Switzerland). All other chemicals and buffer components were obtained from Carl Roth (Karlsruhe, Germany). NunclonTMΔ surface 24 well plates were purchased from Nalgene nunc (Penfield, USA) and oxygen permeable foils (Gas Permeable Adhesive Seals # AB-0718) from Thermo Fisher Scientific (Waltham, MA, USA).

### Cloning and expression of full length and truncated hFMO2

The T1414C mutant of the gene coding for human flavin containing monooxygenase 2 (NCBI accession number BC005894) was ordered at GenScript (NJ, USA). Plasmid DNA was retrieved according to the protocols delivered with the genes and used as templates for gene amplification using Phusion® High-Fidelity DNA polymerase (Finnzymes, Vantaa, Finland) with primers 5′-ATG GCA AAG AAG GTA GCT G-3′ and 5′-AAC TAG GAC CAT TGA AGT TGG C-3′. This insert was A-tailed with Taq polymerase (Thermo Fisher Scientific) and cloned into the pEamTA vector as described previously [16]. The hFMO2 sequence in construct pEamTA:hFMO2 was confirmed by sequencing at LGC Genomics (Berlin, Germany). The construct was then used as a template for the construction of truncated hFMO2 proteins. Specifically, the genes were amplified with Phusion® High-Fidelity DNA polymerase using forward primer 5′-TTA AGC ATA TGG CAA AGA AGG TAG CTG TG-3′ in combination with one of the reverse primers as stated in Table 1. Subsequently, the fragments were ligated into the pMS470 vector via *Nde*I and *Hind*III restriction sites, transformed into electrocompetent *E. coli* TOP10 F’ cells and the sequences were confirmed by Sanger sequencing (LGC Genomics). Finally, electrocompetent *E. coli* BL21 (DE3) Gold cells were transformed with plasmid DNA to give nFMO2 expression strains. The nFMO2 expression strains were cultivated as follows: overnight cultures [20 mL Luria Bertani (LB) medium supplemented with 100 µg/mL ampicillin], inoculated with a glycerol stock sample and grown at 37°C in an orbital shaker) were used to inoculate 500 mL LB/ampicillin medium in 2 l baffled Erlenmayer flasks (OD_600_ = 0.05). These main cultures were grown at 37°C and 110 rpm to an OD_600_ of 0.8 and subsequently induced with 50 µL of IPTG (1 M). After incubation for 24 h at 30°C and 110 rpm, the cells were harvested by centrifugation (4,000*g*, 4°C, 10 min) and the pellets were stored at −20°C.

### Biomass production

Shake flask cultivations were carried out as follows: A glycerol stock of *E. coli* BL21(DE3)/pMS470nFMO2*1 was thawed and 20 µL were used to inoculate 20 mL of LB medium (containing 100 µg mL^−1^ ampicillin) in Falcon tubes, respectively. The precultures were incubated at 90 rpm on a rotary shaker at 37°C overnight. Fresh LB cultures (500 mL) with ampicillin (100 µg mL^−1^) in baffled 2-L Erlenmeyer flasks were inoculated to OD_600_ 0.05 with the preculture. The main cultures were incubated at 37°C with shaking at 110 rpm until an optical density of 0.6–0.8 was reached. FMO2 expression was induced by addition of IPTG (1 mM). The cultures were incubated at 30°C with shaking at 110 rpm for 24 h. Finally, the cells were harvested by centrifugation (10 min, 4,000 rpm). The cell paste (approximately 2 g of wet cells) was suspended in 8 mL of potassium phosphate buffer (50 mM, pH 8.5), and stored at −20°C.

Catalyst preparation in the bioreactor was carried out as follows: Cells of *E. coli* BL21 (DE3) Gold harboring the vector with the FMO2*1X510 gene were taken from vials stored in liquid nitrogen and streaked on LB agar plates containing ampicillin (100 mg/L). The plates were incubated at 37°C for 6.5 h. A loop of the resulting cells was used to inoculate 10 × 500 mL baffled flasks each containing 100 mL LB media supplemented with ampicillin (100 mg/L). Following incubation for 18 h at 30°C with shaking at 200 rpm (orbital shaker, 5 cm radius), the pooled pre-cultures were used to inoculate 100 L of LB/ampicillin (100 mg/L) and Aseol antifoam (0.01% v/v) in a Braun Biotech 150 L fermentation vessel. Fermentation parameters were as follows: temperature 30°C, stirring 150 rpm and airflow 10 L/min. No pH regulation was employed. When OD_600_ reached 0.6–0.8, filter sterilized IPTG was added to a final concentration of 1 mM. After approx. 24 h cultivation, the biomass was harvested by continuous flow centrifugation at 13,000 rpm in a Heraeus Contifuge 20RS at 4°C. The resulting cell paste (340 g) was then shock frozen in dry ice before storage at −80°C.

### Cell fractionation

A cell pellet corresponding to 50 OD_600_ units was resuspended in potassium phosphate buffer (50 mM, pH 8.5) and disrupted by ultrasonication (6 × 30 s). The resulting lysate was centrifuged for 5 min at 5.000×*g* and 4°C to remove cell debris and unbroken cells. In a next centrifugation step (30 min at 10.000×*g* and 4°C) inclusion bodies, if present, were separated. To recover the membrane fractions from *E. coli*, the cleared lysates were ultra-centrifuged at 100.000×*g* and 4°C for 1 h. The resulting supernatant contained the cytosolic protein fraction.

Total protein concentrations of each fraction were determined by the BCA protein assay (Thermo Fisher Scientific), according to the manufacturer´s instructions, using bovine serum albumin as standard.

### Western blot analysis

50 µg of total protein per lane was separated by SDS-PAGE under reducing conditions using NuPAGE® 4–12% Bis–Tris gel (Life Technologies, Carlsbad, USA). Protein bands were transferred onto a nitrocellulose membrane (GE Healthcare, Chalfont St Giles, UK) electrophoretically in a wet blotting system. Immunoblot detection was performed using an FMO2-specific antibody (Abcam, Cambridge, UK, ab171907) according to the manual provided by the supplier. The presence of FMO2 was visualized by staining with nitro blue tetrazolium/5-bromo-4-chloro-indolylphosphate (NBT/BCIP; Merck, Darmstadt, Germany).

### Resting cell biotransformation

Frozen cell pellet was thawed and suspended in reaction buffer (50 mM potassium phosphate buffer, pH 8.5). The cell suspension was equally divided into 24 well plates to give an OD_600_ of approximately 15 based on the final volume of 1 mL. The reaction was started by addition of 10 µL MgCl_2_ (1 M), 50 µL NADP^+^ (1 mM), 50 µL trisodiumcitrate (1 M) and 10 µL substrate in MeOH (100 mM). The plate was sealed with oxygen permeable foil and agitated at 37°C for 16 h on a Titramax (900 rpm). 500 µL samples were drawn and mixed with 500 µL MeOH thoroughly. After centrifugation, the supernatant was analyzed by HPLC/MS. The optical density of the remaining sample was measured in order to determine a correction factor.

### Analysis by HPLC–MS

For HPLC measurements, an Agilent Technologies 1200 Series equipped with G1379B degasser, G1312B binary pump SL, G1367C HiP-ALS SL autosampler, a G1314C VWD SL UV detector, G1316B TCC SL column oven and a G1956B MSD mass selective detector was used. The analytes were separated on an Agilent Zorbax SB-C18 column (1.8 µm; 4.5 × 50 mm) at 50°C by using aqueous eluent (0.1% formic acid) and ACN at a flow of 1.0–1.2 mL min^−1^ which was split to 0.6–0.8 mL min^−1^ before the mass selective detector.

The following gradients were used to separate substrate and products from whole cell conversions: benzydamine (0–2.10 min: 20–70% ACN, 2.10–2.20 min: 70–90% ACN, 2.20–2.40: 90% ACN, 2.40–2.60 min: 90–20% ACN); ethionamide (0–1.20 min: 0–70% ACN, 1.20–2.10 min: 70% ACN, 2.10–3.00: 70–0% ACN); propranolol (0–3.00 min: 0–100% ACN, 3.00–3.50 min: 100% ACN, 3.50–4.00: 100–0% ACN); trifluoperazine (0–2.50 min: 15–100% ACN, 2.50–3.50 min: 100% ACN, 3.50–3.51: 100–15% ACN).

### Typical example of preparative scale biotransformation

**Figure 1:**
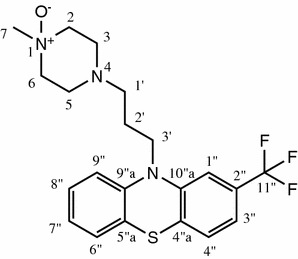
Chemical structure of the identified trifluoperazine metabolite including atom numbering.

Frozen cell paste was thawed at room temperature and re-suspended in a minimal volume of 50 mM potassium phosphate buffer pH 8.5. The suspension was held on ice until required. Preparative biotransformations were carried out in a total volume of 0.2 L phosphate buffer pH 8.5 in a 0.5 L baffled flask containing 3.4 g wet weight *E. coli* cells corresponding to an OD_600_ of 15, 50 mM trisodiumcitrate, 10 mM MgCl_2_, 50 µM NADP^+^ and 100 mg trifluoperazine (final concentration 500 mg/L). The flasks were incubated at 37°C with agitation at 110 rpm on an orbital shaker (5 cm radius). At various time points aliquots were removed and mixed with an equal volume of ACN before centrifuging at 13,200 rpm for 2 min. The supernatants were analyzed using an Agilent Zorbax SB-C18 column (1.8 µm; 4.5 × 50 mm) at 50°C. UV absorption was monitored at 255 nm and the mobile phases consisted of (0.1% formic acid) and ACN at a flow of 1.5 mL min^−1^ (0–2 min: 40–80% ACN). After 22 h incubation, the biotransformation yield of *N*-oxide (Figure 1) reached 89.6%.

The broth was mixed with an equal volume of ethyl acetate and stirred at room temperature for 2 h. The mixture was then centrifuged for 1 h at 10,000 rpm in a Sorvall JA10 rotor at 4°C. The organic phase was collected and the ethyl acetate evaporated under reduced pressure at 40°C. The residue was dissolved in 2 mL dimethylsulfoxide (DMSO) and this solution contained 95 mg *N*-oxide according to HPLC analysis.

The crude product was mixed with 2 mL ACN and 2 mL water and filtered through a PTFE syringe filter. The filtrate was pumped directly onto the RP18 chromatography column concurrently with a 9-fold larger volume stream of an aqueous solution (0.025% v/v) of trifluoroacetic acid (TFA). The conditions for preparative HPLC were: stationary phase: Chromolith prep. RP18e 100 × 25 mm (Merck, 1.25252.0001); solvent A: aqueous TFA 0.025%; solvent B: ACN; gradient: 0–5 min 10% B, 45 min 40% B; flow rate of 50 mL/min; room temperature; detection at 259 nm; fraction size 50 mL. The product eluted between 24 and 25% B. All fractions were analyzed by LC/MS–UV. Fractions containing >95% of product (relative LC/UV peak area) were combined, the solvents were evaporated under reduced pressure to about 50 mL and finally dried by lyophilisation overnight. The product, 48 mg 1-methyl-4-(3-(2-(trifluoromethyl)-10H-phenothiazin-10-yl)propyl)piperazine 1-oxide, was obtained in 46% isolated yield >98% purity (HPLC/full diode array detection) ^1^H and ^13^C NMR spectra were recorded on a Bruker Avance III instrument (^1^H NMR 600.13 MHz, ^13^C NMR 150.9 MHz).^1^H NMR (600 MHz, DMSO-d_6_) δ 7.36 (d, J = 8.0 Hz, 1H, H-4″), 7.28–7.21 (m, 3H, H-8″/1″/3″), 7.18 (d, J = 7.6 Hz, 1H, H-6″), 7.08 (d, J = 8.2 Hz, 1H, H-9″), 7.00 (t, J = 7.5 Hz, 1H, H-7″), 4.19 (t, J = 7.5, 2H, H-3′), 3.75 (m, 4H, H-2/6) 3.53 (s, 3H, H-7), 3.20 (d, J = 12.1 Hz, 2H, H-3), 3.03 (s, 1H, H-5), 2.87 (s, 2H, H-1′), 1.96–1.90 (m, 2H, H-2′).^13^C NMR (151 MHz, DMSO-d_6_) δ ppm, 23.15 (C-2′), 44.61 (C-3′), 46.31 (C-5), 46.56 (C-3), 53.68 (C-1′), 56.59 (C-7), 62.55 (C-6), 62.57 (C-2), 112.39 (C-1″), 116.85 (C-9″), 119.54 (C-3″), 123.48 (C-5″a), 123.71 (C-7″), 127.89 (C-6″), 128.21 (C-4″), 128.40 (C-8″), 129.05 (C-2″), 124.55 (C-11″), 130.01 (C-4″a), 144.24 (C-9″a), 145.81 (C-10″a).

## Results and discussion

### Design of FMO2 truncations

Up to now, functional production of human FMO2*1 was achieved by baculovirus driven expression in Sf9 [9] and *Tricoplusia ni* insect cells [1]. However, these expression systems are not suitable for preparative biotransformations. FMO3 and FMO5 have been successfully expressed in *E. coli* [17–19] in contrast to human FMO2. In our hands, FMO2*1 showed very little expression in *E. coli* BL21 compared to isoforms 3 and 5 [20]. Krueger et al. investigated truncated versions of rabbit FMO2*1 in which the protein was expressed lacking the C-terminal membrane anchor in an attempt to solubilize the protein [21]. Similarly, truncated human FMO3 was expressed in *E. coli*. Whereas in the case of rabbit FMO2*1X510 the protein was found in the membrane fraction, FMO3X516 was reported to be cytosolic [22].

In this study, six truncated versions of FMO2*1 were designed and expressed in *E. coli*. FMO2*1X517 (Δ19) resembles FMO3X516 and according to the membrane domain prediction programs TopRed and MPex the membrane anchor is not fully removed like in FMO2*1X510 (Δ26). In addition to the Δ19 and Δ26 variants, FMO2*1 truncations lacking 31, 36, 40 and 45 amino acids were investigated herein (see Table 1).

**Table 1:** Summary of FMO2 truncation variants generated in the present study. The sequence of the reverse primer to amplify the truncated gene from the full-length template is shown.

FMO2 variant	Reverse primer
FMO2*1X517 (FMO2Δ19)	5′-TTA AGA AGC TTT TAC AAC AGA AAA GAA ACT GAG AAA TTA G-3′
FMO2*1X510 (FMO2Δ26)	5′-TTA AGA AGC TTT TAA TTA GAT GAA TCC TTC AGG GC-3′
FMO2*1X505 (FMO2Δ31)	5′-TTA AGA AGC TTT TAC AGG GCC CGA GTC-3′
FMO2*1X500 (FMO2Δ36)	5´-TTA AGA AGC TTT TAG AGT GGC TTC AGT ATT C-3′
FMO2*1X496 (FMO2Δ40)	5′-TTA AGA AGC TTT TAT ATT CTT TGT TTC TGG GTG AAG-3′
FMO2*1X491 (FMO2Δ45)	5′-TTA AGA AGC TTT TAG GTG AAG ATG GCA TTT C-3′

### Expression and localization of full-length and truncated FMO2*1

To investigate the subcellular location of the generated FMO2*1 truncations, Western blot analyses of cytosolic and membrane protein fractions with FMO2 specific antibodies were conducted.

**Figure 2:**
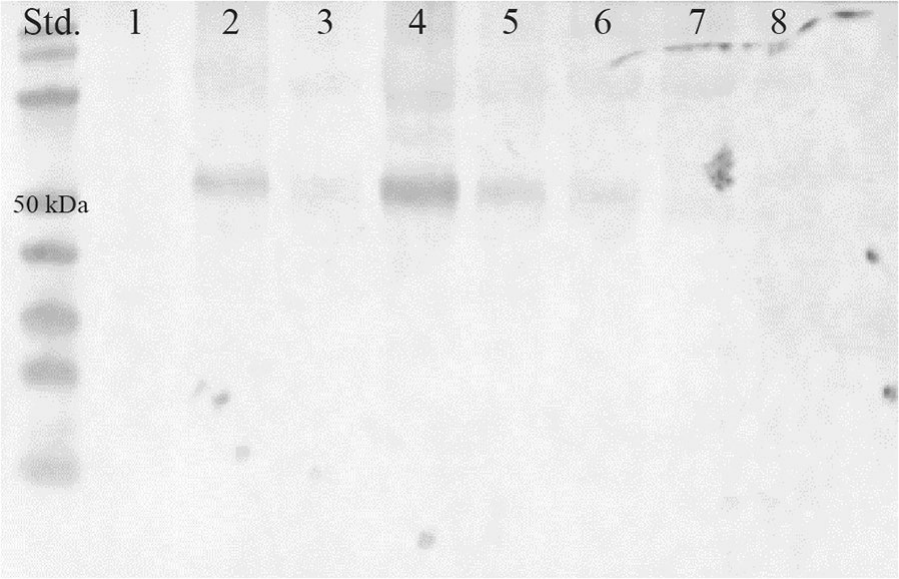
Western blot analysis of full-length and truncated FMO2*1 in the membrane fraction of recombinant *E. coli* strains. 50 µg total protein of the membrane preparation of the empty vector control (pMS470, *1*), FMO2*1 (*2*), FMO2*1X517 (*3*), FMO2*1X510 (*4*), FMO2*1X505 (*5*), FMO2*1X500 (*6*), FMO2*1X496 (*7*), FMO2*1X491 (*8*) were loaded, respectively. (Std.) PageRuler Prestained Protein Ladder (Thermo Fisher Scientific). The blot was probed with anti-hFMO2 antibody.

As shown in Figure 2, removing amino acids from the C-terminus of FMO2*1 did not affect the membrane-bound nature of the corresponding proteins. Like full-length FMO2*1, all the generated FMO2*1 truncations were detected in the membrane fraction, but not in the cytosolic one or in inclusion bodies (data not shown). This is in accord with studies of FMO2Δ64 (FMO2*1X472), the product of the major human FMO2 allele: It was shown that the removal of the 64 amino acid residues had no effect on protein targeting, FMO2*1X472 being still attached to the membranes of the endoplasmatic reticulum in *T. ni* insect cells [1]. Also in the case of rabbit and monkey FMO2, C-terminal truncations did not change the subcellular localization in comparison to the full-length enzyme [12, 23]. These findings and evidence from homologous enzymes [21] suggest that not only the C-terminal part is essential for membrane association. When the amino acid sequence of FMO2*1 is analyzed by transmembrane domain prediction programs (MPEx, Scampi-msa and OCTOPUS), the *N*-terminus is also predicted to be a membrane anchor (amino acid 4-19). The amino acids 4–34 constitute the putative flavin adenine dinucleotide (FAD) binding domain including the GxGxxG motif for FAD-binding [24]. Consequently, *N*-terminal truncation is not an option to solubilize hFMOs.

Although the C-terminal truncations did not result in soluble FMO2 protein, they had a pronounced effect on the levels of recombinant protein as assessed from Western blot analysis (Figure 2). In comparison to the full length protein, the production of the truncation FMO2*1X510 was increased. All other truncated FMO2 proteins were produced in lower amounts, expression levels for the shortest truncations (FMO2*1X496 and FMO2*1X491) being even below the detection limit. As the latter ones also did not show activity in whole-cell conversions of ethionamide (see below), it might be concluded that these variants were not expressed at all. Most probably, removing certain stretches of the protein impairs proper protein folding and in further consequence the protein stability.

### Catalytic activities of full-length and truncated FMO2*1

**Figure 3:**
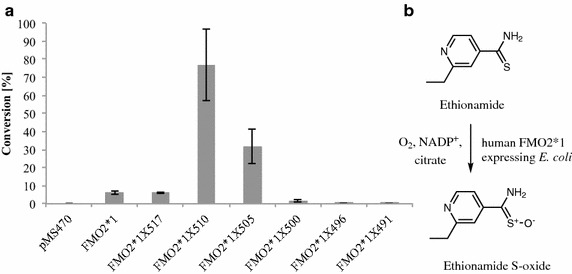
FMO2 based biotransformations of ethionamide. **a** Whole-cell conversions of ethionamide employing *E. coli* catalysts based on FMO2*1 and truncations thereof. The conversions were conducted with whole cells corresponding to 15 OD_600_ units and 1 mM ethionamide for 16 h. Values are shown as mean ± SD of measurements performed in triplicates. Cells carrying the empty vector served as negative control (pMS470). **b** Reaction scheme of the FMO2 catalyzed ethionamide oxidation according to the literature [8, 9].

To investigate the activity of the generated FMO2 catalysts, whole-cell conversions of ethionamide were performed. Ethionamide is a second-line drug for the treatment of tuberculosis. Its antibiotic effect emerges after sulfur oxidation, a reaction that is also catalyzed by human FMO2*1 (Figure 3b) [8, 9]. Expression strains of FMO2*1 and its truncated versions were cultivated in shake flasks and used for whole cell biotransformations of ethionamide.

Prior to the biotransformations described in the following, the previously used conditions [20] were optimized. The most significant parameter appeared to be the amount of biocatalyst: In order to avoid potential limitations of the oxygen supply (which is the co-substrate), decreasing cell densities were studied. Cell densities ≤10 and ≥50 (OD_600_) at reaction start resulted in decreased conversions as compared to ODs of approximately 20. Since the specific productivity is reciprocally correlated to the biocatalyst amount, a cell density of 15 was considered to be optimal for FMO mediated biotransformations and used for all transformations described herein.

*E. coli* whole-cell catalysts were frozen and thawed before use in order to reach a permeabilized state to ensure efficient transport of substrate, product and cofactor. Compared to fully disintegrated cells, the enzymes involved in NADPH formation retain their functionality and may be exploited for cofactor recycling.

The productivity of the strains for ethionamide metabolization was assessed by comparison of product amounts formed after 16 h of reaction time (Figure 3). The biocatalysts based on FMO2*1 and FMO2*1X517 only showed substrate conversions <10%. The ethionamide oxidation was significantly more efficient employing the FMO2*1X510 and FMO2*1X505 catalysts, yielding substrate conversions of 70 and 30%, respectively. Consistent with the low or non-detectable levels of recombinant protein, catalysts based on the shorter FMO2 variants (≤FMO2*1X500) did not show substantial ethionamide conversions (Figure 3a).

### Identification of new hFMO2 substrates

Human pulmonary FMO was almost exclusively described to oxidize sulfur containing compounds to their sulfoxides, whereas their animal model homologues also oxidized nitrogens of various amines. Due to the high similarity between these enzymes, we hypothesized that the substrate scope of human FMO2*1 is likely to be broader than reflected in the literature. A small panel of active pharmaceutical ingredients containing sulfur and nitrogens of different chemical ambience were screened as FMO2*1 substrates. Particularly high activity has been observed with benzydamine, a typical FMO substrate (hFMO1 and hFMO3) [24]. It is a nonsteroidal anti-inflammatory drug with additional analgesic and local anesthetic properties, used in the treatment of mouth and throat infections [25].

**Figure 4:**
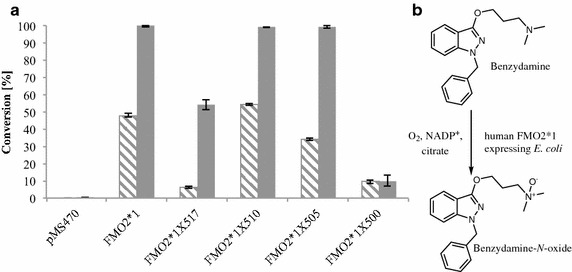
FMO2 based biotransformations of benzydamine. **a** Whole-cell conversions of benzydamine employing *E. coli* catalysts based on FMO2*1 and truncations thereof. The conversions were conducted with whole cells corresponding to 15 OD_600_ units and 1 mM benzydamine for 4 h (*hatched bars*) and 16 h (*filled bars*), respectively. Values are shown as mean ± SD of measurements performed in triplicates. Cells carrying the empty vector served as negative control (pMS470). **b** Reaction scheme of the FMO2 catalyzed benzydamine oxidation. The corresponding *N*-oxide was identified as product by NMR (see Additional file 1).

We incubated benzydamine with resting cells containing FMO2*1. After 16 h of reaction time, 100% conversion was obtained in whole-cell biotransformations employing *E. coli* cells expressing the full-length FMO2*1 protein. Benzydamine has also been reported as a substrate of the FMO2 from cynomolgus macaques, which shares a 98% sequence identity with human FMO2*1 [26]. Interestingly, in contrast to the ethionamide biotransformations, the catalysts based on FMO2*1X510 and FMO2*1X505 did not show an improved performance in comparison to the full-length FMO2*1 catalyst, even at shorter reaction time (4 h). The catalyst FMO2*1X517, although showing the same conversions rates for ethionamide as FMO2*1, displayed significantly less activity in the case of benzydamine (Figure 4). The detected metabolite was the product of a single oxidation as assessed by mass selective detection. Benzydamine contains three nitrogen atoms which raised the question of regioselectivity. One of the three nitrogens in benzydamine is part of an imidic acid functionality and less likely to be oxidized by FMOs compared to the other two tertiary amines. On the one hand, dimethylaniline was found to be oxidized by rabbit FMO2 [10], indicating that tertiary amines are FMO2 substrates. Imipramine and chlorpromazine bear dimethylated amines on an aliphatic chain and perfectly resemble benzydamine in the respective part of the molecule, but these two compounds were described as non-substrates of pulmonary FMO [10, 27]. In literature, indirect methods such as oxygen or NADPH consumption assays were typically used to determine whether a compound is a substrate or not, but these methods do not allow assignment of the site of oxidation. Structure elucidation of the metabolites is indispensable in order to understand the true substrate specificity of metabolizing enzymes, and, in the long run, to be able to predict the metabolites of new xenobiotics [28]. We up-scaled the screening reaction and used 50 mg of benzydamine as a substrate for FMO2*1X510. The crude product was analyzed by NMR (Additional file 1). The dimethylated amine had been oxidized to the respective *N*-oxide, as judged by a shift of the methyl signals and the methylene adjacent to the aliphatic nitrogen. By contrast, lidocaine, which is a diethyl amine, was not a substrate under the screening conditions used herein.

Another compound, which we found to be metabolized by hFMO2, was propranolol. Propranolol is used as a β-adrenergic blocking agent for the treatment of angina pectoris, hypertension and abnormal heart rhythms. The role of propranolol metabolism with cytochrome P450 enzymes (CYP) is well studied whereby the naphthaline part of propranolol is oxidized by CYP2D6 or *N*-dealkylation occurs [29, 30]. In contrast, little attention has been paid to FMO mediated propranolol metabolism. *N*-oxidation of this compound was only described for hepatic porcine FMO [31], but not for the pulmonary FMO isoenzyme until now. We detected significant conversion of propranolol to a product mixture consisting of at least four compounds. Each compound showed the mass of a mono-oxidation product. Our attempts to elucidate the chemical structure of the major metabolite by NMR were unsuccessful due to its decomposition in the NMR solvent. The spectra indicated, however, that *N*-hydroxylamine propranolol was not the primary product. Based on mass selective detection, also *N*-dealkylation can be ruled out and nitrone formation seems unlikely. As described by Baughman et al. for propranolol conversions with hepatocytes of various species [32], the isopropyl part can be oxidized and one could speculate that this reaction may have been catalyzed by FMO2. It remains to be clarified if the product mixture was a result of low metabolite stability in solution, rearrangement reactions or FMO2 selectivity towards propranolol.

### FMO2 based metabolite production on multi mg-scale

**Figure 5:**
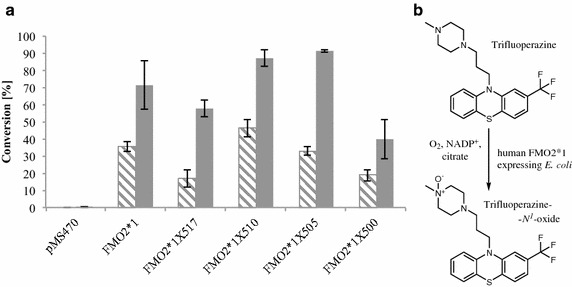
FMO2 based biotransformations of trifluoperazine. **a** Whole-cell conversions employing *E. coli* catalysts based on full-length FMO2 and truncations thereof. The conversions were conducted with whole cells corresponding to 15 OD_600_ units and 1 mM trifluoperazine for 4 h (*hatched bars*) and 16 h (*filled bars*), respectively. Values are shown as mean ± SD of measurements performed in triplicates. Cells carrying the empty vector served as negative control (pMS470). **b** Reaction scheme of the FMO2 catalyzed trifluoperazine oxidation. The corresponding *N* ^1^-oxide was identified as product by NMR (see Additional file 1).

Screening for compounds that are targeted by FMO2 in human drug metabolism further revealed trifluoperazine as an hFMO2 substrate. Trifluoperazine is a member of the phenothiazine family of drugs and it has one sulfur and three nitrogen atoms in its chemical structure. The FMO2 expressing strains described herein exhibited excellent activities for the oxidation of this drug to a single, mono-oxidized metabolite (Figure 5). Based on literature knowledge, the prediction whether the sulfur or one of the three nitrogens had been oxidized was not trivial, because human FMO2 has—to this end—been described as sulfur oxidizing (thioureas, thioetherorganophosphates, thiazetazone, ethionamide [6–9]). By contrast, FMO2 homologues from other mammals were shown to act on amines [10–13]. Poulson et al. ascribed the oxidation of three perazine derivatives by rabbit FMO2 to *N*-oxidation, however, the specific site of oxidation was not elucidated [10].

In order to unambiguously determine the chemical structure of the trifluoperazine metabolite produced by FMO2*1 and its truncated versions, 50 mg of the compound were subjected to biooxidation using *E. coli* cells grown in a bioreactor at the 100 L scale and the product was isolated and characterized by NMR. Compared to the parent compound trifluoperazine, the spectra of the phenothiazine and propane part remained identical in the metabolite, which ruled out sulfur and phenothiazine nitrogen oxidation. However, there was a significant change in the piperazine part of the metabolite. Whereas the ring-methylene groups in trifluoperazine gave a very broad and undefined peak due to the high flexibility of the alicyclic ring, the metabolite signals were defined well. Also peak shifts that are similar to those seen between *N*-methyl-morpholine and its *N*-oxide indicate *N*-oxidation of the piperazine ring. Summarizing, these spectra show that trifluoperazine had been oxidized at the methylated nitrogen of the piperazine ring. Chemical oxidation of trifluoperazine with, e.g. hydrogen peroxide or sodium periodate at room temperature lead to a product mixture without even traces of the human metabolite (data not shown), which is in accordance to a literature report [33]. In contrast, recombinant FMO2 leads to fully chemo- and regioselective oxidation in quantitative yield. This result underlines the importance of having tools available allowing for the simple and straightforward synthesis of authentic human drug metabolites.

## Conclusions

Human flavin monooxygenase 2 (hFMO2) was successfully used in form of *E. coli* based whole-cell catalysts. Solubilizing membrane associated enzymes by removal of their membrane anchor(s) is a strategy to afford higher enzyme levels. In case of hFMO2, *N*-terminal truncation would remove the FAD binding site and C-terminal truncation did not affect protein localization, as reported herein. Although hFMO2*1X510 remained attached to the membrane fraction, a positive effect on its expression level translated in a whole-cell biocatalyst with increased activity. The thus generated catalysts were used on the one hand to investigate the substrate scope of the FMO2 enzyme beyond literature known substrate structures. On the other hand, the synthetic utility of recombinant human FMO2 for metabolite preparation was finally demonstrated by the selective oxidation of a substrate with several soft nucleophiles (trifluoperazine).

## Additional files

Additional file 1:
**Figure S1**. ^1^H NMR of trifluoperazine metabolite; **Figure S2**. 2D HSQC NMR of trifluoperazine metabolite; **Figure S3**. 2D HMBC NMR of trifluoperazine metabolite; **Figure S4**. ^1^H NMR of benzydamine metabolite and benzydamine; **Figure S5**. Comparison of DEPT NMR of benzydamine metabolite with ^13^C NMR of parent benzydamine.

## Abbreviations

ACN: acetonitrile
DMSO: dimethylsulfoxide
FAD: flavin adenine dinucleotide
FMO2: flavin monooxygenase isoform 2
hFMO: human flavin monooxygenase
HPLC: high performance liquid chromatography
IPTG: isopropyl-β-d-thiogalactoside
LB: Luria-Bertani
LC: liquid chromatography
NMR: nuclear magnetic resonance
