# Supplementary material for: Human FMO2-based microbial whole-cell catalysts for drug metabolite synthesis
Source: Microb Cell Fact. 2015 Jun 12;14:82. doi: 10.1186/s12934-015-0262-0 (PMC4464233; doi:10.1186/s12934-015-0262-0)
Supplement: Additional file 1: — Figure S1. 1H NMR of trifluoperazine metabolite; Figure S2. 2D HSQC NMR of trifluoperazine metabolite; Figure S3. 2D HMBC NMR of trifluoperazine metabolite; Figure S4. 1H NMR of benzydamine metabolite and benzydamine; Figure S5. Comparison of DEPT NMR of benzydamine metabolite with 13C NMR of parent benzydamine. [file 12934_2015_262_MOESM1_ESM.docx]

**Supporting Information**

***Human FMO2-based microbial whole-cell catalysts for drug metabolite synthesis***

Martina Geier, Thorsten Bachler, Steven P. Hanlon, Fabian K. Eggimann, Matthias Kittelmann, Hansjörg Weber, Stephan Lütz, Beat Wirz, Margit Winkler

^1^H NMR of trifluoperazine metabolite 2

2D HSQC NMR of trifluoperazine metabolite 3

2D HSQC NMR of trifluoperazine metabolite 4

^1^H NMR of benzydamine metabolite and benzydamine 5

Comparison of DEPT NMR of benzydamine metabolite with ^13^C NMR of parent benzydamine 6

Figure 1 ^1^H NMR of trifluoperazine metabolite

Figure S2 2D HSQC of the trifluoperazine metabolite (see page 2 for compound structure and numbering)

Figure S3 2D HMBC of the trifluoperazine metabolite (see page 2 for compound structure and numbering)

Figure S4 Comparison of ^1^H NMR of benzydamine metabolite with parent benzydamine

Figure S5 Comparison of DEPT NMR of benzydamine metabolite with ^13^C NMR of parent benzydamine
